# Richter Syndrome

**DOI:** 10.1007/s11912-020-01001-x

**Published:** 2021-02-12

**Authors:** Adalgisa Condoluci, Davide Rossi

**Affiliations:** 1 Division of Hematology, Oncology Institute of Southern Switzerland, Bellinzona, Switzerland; 2 Laboratory of Experimental Hematology, Institute of Oncology Research, Bellinzona, Switzerland

**Keywords:** Richter syndrome, CLL, Transformation, DLBCL, Hodgkin lymphoma

## Abstract

**Purpose of Review:**

Richter syndrome (RS) is an uncommon but aggressive evolution of chronic lymphocytic leukemia/small lymphocytic lymphoma (CLL/SLL). RS is an unmet clinical need in the field of CLL. Recent advances in understanding the biology of this condition provide the rationale for testing new therapeutic concepts in order to improve the outcome of patients developing RS, which is so far poor. In this review, we summarize disease characteristics and available therapeutic options for RS.

**Recent Findings:**

Current regimens with novel agents in monotherapy have shown little impact on survival. Nevertheless, the better reported outcome for RS has been achieved with the combination of chemo-immunotherapy with a novel agent, confirming the synergistic effect of the approaches. Still, the frailty of this population may impose a less toxic management leaving most patients with no reasonable therapeutic option.

**Summary:**

Treatment options for RS need to be further expanded. Preclinical models in current development may allow to explore actionable pathways and identify new drug targeted combinations.

## Introduction

The transformation of CLL or SLL into an aggressive lymphoma was firstly described in 1928 as a “reticular cell sarcoma” by Maurice Richter [1] and then nominated in his honor as Richter syndrome. As described within the WHO Classification, RS may present as two different pathologic entities: the diffuse large B cell lymphoma (DLBCL) variant or, rarely, the Hodgkin lymphoma (HL) variant [2].

Some clinical clues of transformation (development of new B symptoms, asymmetric arise of bulky lymph nodes with/without associated organ dysfunction from invasive or obstructive neoplastic growth, and/or sudden rise of lactate dehydrogenase (LDH) levels) should promptly rise suspicion of this life-threatening complication in a patient with CLL. This rare evolution is estimated to occur in 0.5–1% of patients with CLL/SLL per year [3]. Different genetic characteristics explaining the aggressiveness and chemorefractoriness of RS have been identified, including *TP53*, *NOTCH1*, *MYC*, and *CDKN2A* mutations or disruptions [4–6]. Only a fraction of RS (~ 20% with DLBCL morphology and ~ 70% with HL morphology) harbors distinct IGHV-D-J rearrangements compared to the preceding CLL, representing de novo lymphomas developing in a CLL patient [6, 7].

The DLBCL variant of RS is associated with a dismal prognosis with a median survival of < 1 year [6, 8, 9•]. Treatment options commonly used in this setting are based on regimens of de novo DLBCL. However, the limited efficacy obtained with conventional treatments led to the consolidation strategy of stem cell transplantation (SCT) in selected patients. The HL variant shows in contrast better survivals when treated with HL regimens. Several studies evaluating the role of novel agents are ongoing in RS, showing promising benefits when combined with conventional chemo-immunotherapy.

## Morphology and RS Subtypes

### DLBCL Variant

The DLBCL variant is described in approximately 90% of RS. The morphology of the DLBCL variant of RS is characterized by confluent sheets of large neoplastic B lymphocytes resembling either centroblasts (60–80% of cases) or immunoblasts (20–40% of cases) [1, 6, 10]. CLL transformation should be differentiated from CLL progression, which can be associated with the expansion of the proliferation centers in the lymph nodes with confluent and enriched proliferating cells [1]. These forms of “aggressive” CLL or “accelerated” CLL have an outcome intermediate between typical CLL and classic RS. Though not clearly defined by the current WHO Classification of Tumors of Hematopoietic and Lymphoid Tissues, some morphologic criteria have been proposed to correctly distinguish RS from an “accelerated CLL”: (i) tumor of large B cells with nuclear size equal or larger than macrophage nuclei or more than twice a normal lymphocyte and (ii) diffuse growth pattern of such large cells (not just presence of small foci) [11, 12]. The immune phenotype of tumor cells invariably express CD20, while CD5 expression is maintained only in a fraction (~ 30%) of cases, and CD23 expression is even more rare (~ 15% of cases) [7]. PD-1 expression, described only on the paraimmunoblasts of proliferation centers, is common in DLBCL variants, whereas it is only rarely found in de novo DLBCL specimens.

Based on the analysis of the rearrangement of IGHV-D-J genes, most (~ 80%) of the DLBCL variants of RS are clonally related to the preceding CLL phase, thus representing true transformations [5, 7]. This information profoundly impacts on prognosis, with clonally related cases having a median survival of approximately 12 months, while clonally unrelated RS show a similar survival to DLBCL de novo cases (nearly 65 months).

### HL Variant

The presence of classical Reed-Sternberg cells harboring a CD30-positive/CD15-positive/CD20-negative phenotype in a proper polymorphous background of small T cells, epithelioid histiocytes, eosinophils, and plasma cells defines the HL variant of RS [10]. This variant accounts for only 5–10% of RS. Only a fraction (~ 30%) of the HL variant of RS are clonally related to CLL [10], while most cases (65–75%) are EBV positive with distinct immunoglobulin rearrangements compared to the paired CLL, thus representing de novo, EBV-driven lymphomas arising in a CLL patient [10].

## Epidemiology, Genetics, and Risk Factors

Prevalence of DLBCL variant RS is highly variable (1–23%) and depends on a number of factors: (i) whether the analysis is restricted to biopsy-proven cases or also includes patients with clinically suspected transformation; (ii) the diagnostic aggressiveness in case of rapidly progressive lymphadenopathy; and (iii) in the set of clinical trials, the selection of patients who fit the eligibility criteria for trial participation, and in which the therapy used may have influenced the risk of transformation. The prevalence of RS in a large cohort (*n* = 2975) of prospectively monitored patients with advanced CLL enrolled in trials of the GCLLSG was 3% [13•]. Transformation can occur early after the diagnosis of CLL, with a reported median time to transformation of 1.8–1.9 years for DLBCL [3, 14] and 4.6–7.5 years for HL [15, 16], with a fraction of patients never being treated for CLL before the transformation.

**Fig. 1:**
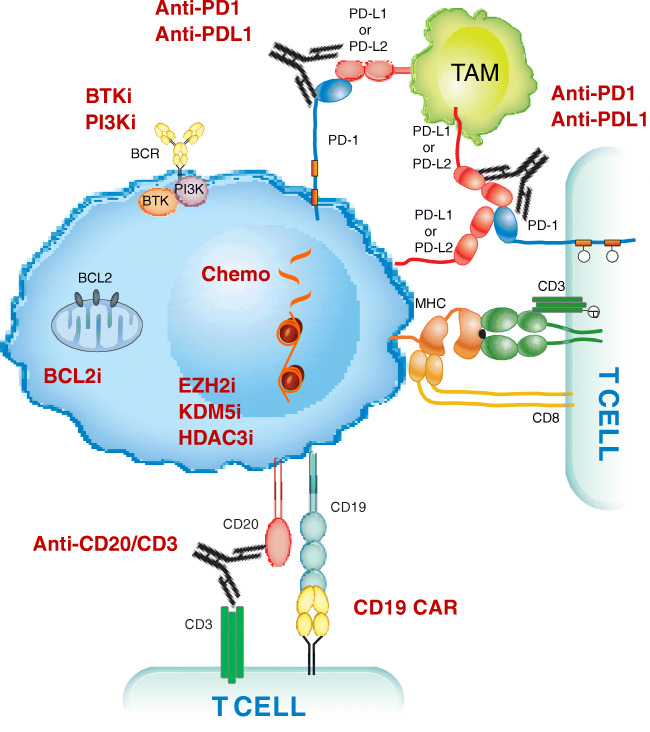
Transformed lymphoma vulnerabilities and targets for treatment. A representation of the complex process and molecular pathogenesis of transformed lymphomas, resulting from a number of epigenetic and genetic lesions occurring in the tumor cell population (reported in black). Non-genetic mechanisms as pathway activation and changes in immune checkpoints profile are also involved in transformation. Communication between the tumoral cells (in blue) and T cells is established by direct contact, chemokine/cytokine receptors, adhesion molecules and ligand-receptor interactions. Environmental or auto-/self-antigens and homotypic IG interactions trigger BCR activation, which stimulate underlying CLL proliferation. Immune inhibitory molecules (PD-L1 among others) facilitate tumor cells to evade immune-response and maintain tolerance. Recurrently mutated genes in RS affect DNA repair, B cell receptor, and chromatin modification. Potential and established targeted treatments in RS are reported in red. TAM, tumor associate macrophage; BCR, B cell receptor

The genetics of the DLBCL variant is different from that of a de novo DLBCL, lacking molecular lesions in signaling pathways and B cell differentiation programs, while sharing with other transformed lymphomas (i.e., transformed follicular lymphoma) lesions affecting general regulators of tumor suppression, cell cycle, and proliferation (Fig. 1) [4–6]. Somatic mutations of *TP53*, *NOTCH1*, *MYC*, and *CDKN2A* account for the aggressive phenotype of the DLBCL variant, which combines chemoresistance and rapid disease kinetics (Fig. 1) [4–6].

*TP53* is the most frequently disrupted gene in the DLBCL variant, acquiring at the time of transformation either mutation or deletion in ~ 60% of cases. Being a master regulator of the DNA-damage-response pathway which leads to cell apoptosis if activated (i.e., as in response to the antiproliferative effect of chemotherapies), *TP53* loss may explain the chemorefractory phenotype generally shown by RS. *CDKN2A,* which can be found deleted in 30% of cases, is a negative regulator of cell cycle transition from G1 phase to S phase [5, 6]. Cell cycle deregulation by *CDKN2A* may explain the rapidly progressive behavior of DLBCL variant. *MYC* genetic alterations sustain ~ 40% of DLBCL variant [5, 17]. *MYC* is involved in a transcription regulating network which is generally deregulated by somatic structural alterations of this gene in ~ 30% of cases [4–6, 14]. The usage of the subset 8 configuration in the B cell receptor (BCR), which has been reported to have an association to *NOTCH1* somatic mutations and which shows an unlimited propensity to autonomous BCR signaling and to respond to multiple auto-antigens and immune stimuli from the microenvironment, may explain the particular aggressiveness of CLL harboring subset 8 BCR and their increased propensity to transform into RS [10, 18]. In one study, the 5-year transformation rate of patients with CLL and subset 8 usage has been reported at nearly 70% [10]. The mutational status of *NOTCH1* is the only validated risk factor for transformation, with a significantly higher cumulative probability of patients with CLL developing DLBCL variant (45%) compared to CLL without *NOTCH1* mutations (4%) [19–21].

EBV infection has been suggested as a pathogenetic trigger of DLBCL variant RS. The observation that the overwhelming majority (85–100%) of DLBCL transformed from CLL does not carry EBV infection in the malignant cells, however, does not favor this hypothesis [6].

The role of the exposure to a prior CLL treatment as a risk factor for transformation is controversial, and a proportion of patients who develop RS show no prior therapy for the underlying CLL. In this setting, better outcomes have been reported (median overall survival 35 months for treatment-naïve CLL patients vs 4 months for previously treated in one report [22•]; 46 months vs 7 months, respectively, in one report [9•]). The effect of novel agents on RS development is progressively being reported. Clonal evolution leading to transformation seems to be similar in the novel agent treatment setting to that of chemo-immunotherapy (CIT), with frequent associations of *MYC*, *CDKN2A*, *TP53*, and *NOTCH1* disruption [23••, 24••].

While novel agents do not seem to increase the proportion of RS, transformation rates of 5–16% in high-risk and heavily pretreated patients in study population on novel agents have been reported. In this setting, RS occurs typically within the first 18 months of treatment, with a median OS of approximately 6 months after transformation [25].

Early recognition of RS transformation helps to avoid the exposure of patients to multiple lines of therapy that, being targeted to CLL progression, are of little efficacy for the transformed clone. This concept prompts the need for a close monitoring of CLL patients harboring risk factors of RS development.

## Diagnosis

Patients with known CLL developing physical deterioration, fever in the absence of infection, rapid and discordant growth of localized lymph nodes, and/or sudden and excessive rise in lactate dehydrogenase (LDH) levels should be suspected for Richter transformation. Likewise, in the case of extra-nodal masses developing in patients with a known CLL, RS might be included in the differential diagnosis. Nonetheless, the specificity of these clinical findings for transformation is only 50–60%, with the remaining cases showing either progressive or “accelerated” CLL, or even a solid cancer [26].

Histologic documentation, with an open biopsy considered as gold standard for RS diagnosis is mandatory to diagnose RS. Samples obtained with fine needle biopsy or aspiration may not be representative of the pathologic architecture of the tumor, with possible false-positive diagnosis (i.e., fine needle biopsy of an enlarged proliferation center, which may be occasionally observed in lymph nodes of progressive or “accelerated” CLL) [27]. Since RS is often restricted to one single lesion at transformation, any biopsy aimed at exploring whether RS has occurred should be directed at the index lesion.

The ^18^FDG PET/CT has an established role in supporting the choice of whether to perform a biopsy and may tailor the biopsy to the likely transformed site since sites affected by RS are expected to have SUVs overlapping with those of de novo DLBCL [26, 28, 29]. The high negative predictive value (97%) of ^18^FDG PET/CT when a standard uptake value (SUV) cutoff of < 5 is chosen supports a non-biopsy approach suggesting that a transformation is not likely. Conversely, because of the limited positive predictive value (53%) of ^18^FDG PET/CT with an SUV ≥ 5, a biopsy should be directed at the index lesion (i.e., the lesion showing the most avid ^18^FDG uptake, the lesion with the largest diameter by imaging, and/or the lesion showing the most rapid kinetics of progression) [26, 28, 29].

A sensitivity of 91% and a specificity of 95% have been recently reported when using an SUV cutoff of 10, with positive and negative predictive values of 60.6% and 99.2%, respectively [30]. The same report shows a better proportion of correctly classified patients as RS with 94.6% when choosing an SUV cutoff of 10, compared to 73.5% when using an SUV cutoff of 5. Moreover, a better correlation with outcomes has been reported, with median OS of 6.9 months for patients with lesions with an SUV value ≥ 10 compared to 56.9 months for patients with lesions with an SUV < 10. In the largest series of PET/CT prospectively performed in patients following kinase inhibitor discontinuation [31], a SUV threshold ≥ 10 showed low positive (63%) and negative (50%) predictive values. Considering that, the threshold of 10 did not turn out to be a useful noninvasive method to rule out RS post-kinase inhibitor therapy. In the same study, 5 of 8 of the biopsy-confirmed RS showed a SUV ranging from 5 to 9, whereas only 3 of 8 RS had a SUV ≥ 10, further reinforcing the notion that a lower threshold (i.e., SUV 5) should also be used in the setting of kinase inhibitor failure to rule our RS [31].

## Prognosis of RS

The prognosis of DLBCL variant of RS is poor. Based on the number of presenting risk factors (Zubrod performance status > 1, LDH levels above normal values, platelet count ≤ 100 × 10^9^/L, tumor size ≥ 5 cm, and > 2 prior lines of therapy), a validated RS prognostic score segregated 4 risk groups: low risk showing a median survival of 13–45 months (0–1 risk factors); low intermediate risk with a median survival of 11–32 months (2 risk factors); high intermediate risk showing a median survival of 4 months (3 risk factors); and high risk with a median survival of 1–4 months (4–5 risk factors) [32]. The most influent prognostic factor is the clonal relationship between the transformed DLBCL and the underlying CLL. Indeed, patients with a clonally unrelated DLBCL show a longer median survival (5 years) compared with patients with a clonally related DLBCL transformation (8–16 months) [6, 22]. RS after ibrutinib or venetoclax shows an even more aggressive behavior. Outcomes are generally poor for patients with a refractory disease, which overall accounts for more than 80% of cases [32–37].

## Treatment Options of the DLBCL Variant of RS

### Chemo-immunotherapy Approach

Regimens indicated for aggressive B cell non-Hodgkin lymphomas have been proposed to treat patients developing the DLBCL variant of RS. The choice of treatment for patients presenting RS needs to be evaluated in view of their history and comorbidities.

The reported response rate of 8 courses of R-CHOP (rituximab, cyclophosphamide, doxorubicin, vincristine, and prednisone) is 67% (complete response, CR 7%), with a median progression-free survival (PFS) of 10 months and a median overall survival (OS) of 21 months. Hematotoxicity is reported in 65% of patients, while infections are the most common severe non-hematologic toxicity in 28% of patients [38]. In a retrospective series of 48 patients treated with R-CHOP, the overall response rate (ORR) was 37%, with a median OS of 35 months [22•].

Ofatumumab (O), an anti-CD20 monoclonal antibody with greater complement-mediated cytotoxicity than rituximab, combined with CHOP showed an ORR of 46% (CR 27%, PR 19%) with a median PFS of 6 months and a median OS of 11 months. Adverse events under CHOP-O were infections and hematologic toxicities (thrombocytopenia, febrile neutropenia, sepsis) [39, 40].

R-EPOCH (rituximab, etoposide, prednisone, vincristine, cyclophosphamide, and doxorubicin) is used in high-grade B cell lymphoma with rearrangements of *MYC* and *BCL2* and/or *BCL6* (double-hit and triple-hit lymphomas). Since *MYC* is frequently rearranged in the DLBCL variant of RS, R-EPOCH has been investigated in this disease as first-line therapy showing a 20% response rate, a median PFS of 3 months, and a median OS of 6 months [41]. Characteristics of underlying CLL influenced outcomes of R-EPOCH, with worse PFS and OS in deletion 17p and complex karyotype patients. Main adverse events were due to hematologic toxicities (febrile neutropenia and infections) [41].

The hyper-CVAD regimen, a fractioned cyclophosphamide, vincristine, doxorubicin, and dexamethasone regimen, alone or in alternating combination with methotrexate and ara-C, resulted in response rates of ~ 40% with poor median OS. These aggressive regimens were invariably complicated by severe hematotoxicity in all cases, translating into a high severe infection rate of 50% and a treatment-related mortality of ~ 20% [42] despite the prophylaxis with granulocyte-macrophage colony stimulating factor (GM-CSF) [43].

Platinum-containing regimens with the combination of oxaliplatin, fludarabine, ara-C, and rituximab have been explored within the OFAR 1 and OFAR 2 trials. The ORR of OFAR 1 trial was 50% (CR 6–20%), though with short duration of response (mean PFS of 3 months and mean OS of 6–8 months) and severe myelosuppression [44]. The OFAR 2 trial, designed with the aim of improving clinical outcomes and decreasing toxicities with modification of oxaliplatin and cytarabine doses, did not show actual improvement of toxicity rates with 80% of patients developing grade 3–4 neutropenia/thrombocytopenia and 20% grade 3–4 infections. The ORR was 39% (CR 6.5%); the median PFS was 3 months; the median OS was 7 months; and at 2 years, only 19.7% of patients with RS were alive [45].

### Radioimmunotherapy

No responses have been documented in 7 RS patients treated with radio-immunotherapy in a single institution trial investigating ^90^Y ibritumomab tiutexan, with 100% of progression at a median time of 40 days [46, 47].

### Role of Stem Cell Transplantation Consolidation

Due to the unsatisfactory durability of response after chemo-immunotherapy, SCT has been explored as post-remission therapy in RS fit patients. However, only 10–15% of patients with RS can access SCT generally due to their frailty (age, performance status) and donor availability [48].

The efficacy of SCT in RS is granted by dose intensity delivered by high-dose cytotoxic therapy and, in the case of allogeneic SCT, graft-versus-leukemia activity. Indeed, in patients undergoing autologous SCT, no clear plateau in relapse-free survival is described, but only a fraction of relapses seems related to RS, while the remainder are due to CLL. This data suggests that autologous SCT may be efficacious on the eradication of the RS component but not on the underlying CLL component. The plateaus of relapse-free survival among RS patients treated with reduced intensity conditioning (RIC) allogeneic SCT support the presence of a graft-versus-leukemia effect in RS [48].

The benefit of receiving SCT is reported as a longer median survival (5 years vs < 1 year for patients not receiving SCT) [48]. At 3 years, the survival after allogeneic SCT was 36% and 59% after autologous SCT, with a respective relapse-free survival of 27% and of 45%. The non-relapse mortality at 3 years was 26% after allogeneic SCT and 12% after autologous SCT [48].

The main factor influencing the post-transplant outcome is disease status at SCT. Indeed, patients who undergo SCT with chemotherapy-sensitive RS had a superior survival compared to those who undergo transplantation with active and progressive disease. The major benefit of SCT was obtained in young (< 60 years) patients. Among patients receiving allogeneic SCT, those conditioned with a reduced intensity regimen had the longest survival [48].

A recent analysis from the German CLL Study Group showed a median OS of 17 months in 3 patients undergoing allogeneic SCT for RS [13•].

A meta-analysis assessing the efficacy of allogeneic SCT for RS patients reported a relapse rate of 28% with a non-relapse mortality of 24% which is in line with previous reports on lymphoid malignancies [49].

Overall, these data suggest that both autologous SCT and reduced intensity conditioning allogeneic SCT can be effective in young patients with a chemosensitive RS. For patients suitable to transplant but lacking a donor, autologous stem cell transplantation may be an alternative option.

### HL Variant

In the setting of the HL variant RS, the response rate of ABVD (doxorubicin, bleomycin, vinblastine, dacarbazine) is 40–60%, with a median overall survival of 4 years. Indeed, the standard of care for de novo HL is the most frequently used regimen for patients with the HL variant of RS. [50–53]. ABVD is associated with the risk of serious pulmonary toxic effects due to the bleomycin exposure [53]. Applying the results from the advanced stage HL trials, bleomycin can be omitted after two cycles of ABVD if interim PET shows negative Deauville score (score 1–3). Escalation to BEACOPP in fit and younger patients might be considered in case of a positive interim PET. For older and unfit patients, the addition of radiotherapy could be an option [54•]. Stem cell transplantation is less used for consolidation in this setting, because of the longer survival observed compared to the DLBCL variant.

### Novel Agents in RS

Transformed lymphomas show common molecular signatures presenting deregulation of tumor suppression, cell cycle and proliferation pathways [7, 55]. Recent studies have revealed the molecular pathogenesis of transformed lymphomas including RS, showing a complex process, resulting from a number of epigenetic and genetic lesions occurring in the tumor cell population. Non-genetic mechanisms as pathway activation and changes in immune checkpoints are also involved in transformation (Fig. 1). This novel knowledge encouraged clinical investigations on a variety of targeted therapeutic strategies (Table 1), also prompted by the unsatisfactory response rates obtained with conventional chemo-immunotherapy associated to a short response duration without a SCT for consolidation, which cannot be proposed to the majority of RS patients because of the constrains imposed by a combination of age, poor performance status, lack donor availability and refractoriness to induction treatments.

**Table 1:** Ongoing trials in Richter syndrome

Interventions	Ref.
Acalabrutinib+R-CHOP	NCT03899337
Venetoclax+DA-EPOCH-R	NCT03054896
Ibrutinib+Nivolumab	NCT02420912
Zanubrutinib+Tislelizumab	NCT04271956
Duvelisib+Nivolumab	NCT03892044
Copanlisib+Nivolumab	NCT03884998
Duvelisib+Venetoclax	NCT03534323
Umbralisib+Ublituximab	NCT02535286
Atezolizumab+Obinutuzumab+Venetoclax	NCT02846623
Atezolizumab+Obinutuzumab+Venetoclax	NCT04082897

The nucleo-cytoplasmic transport of proteins is often misregulated in cancer and depends on the activity of export proteins, including XPO1 which transports tumor suppressor proteins. An increased activity of nuclear exportation, with a related inhibition of the physiologic tumor-suppressor processes, is often observed in tumoral diseases. Selinexor is a selective inhibitor of nuclear export aiming at retaining tumor suppressor proteins in the nucleus, thus activating them in tumor cells. In a phase I study, selinexor showed signal of activity in 33% of the patients with the DLBCL variant of RS [56]. Few grade 3–4 adverse events were reported (5%) [56]. The phase 2 study (NCT02138786) has been terminated early, due to enrollment challenges.

Bruton’s tyrosine kinase (BTK), a component of the B cell receptor (BCR) signaling pathway, is a strong regulator of cell proliferation and survival in B cell malignancies. Targeted BTK inhibition is described to act in CLL with growth inhibition and cell death by blocking BCR-induced BTK activation [57, 58]. This activity is maintained in patients with high-risk disease (i.e., CLL with *TP53* disruption). In a study of four patients with RS, responses in three patients, including one CR and two partial responses (PRs), were reported [59]. Other case studies reported responses in patients with DLBCL variant RS on ibrutinib [60, 61], with PFS of up to 16 months [61].

Acalabrutinib is a second-generation oral BTK inhibitor which selectively and irreversibly binds cysteine residues on BTK [62]. In the ACE-CL-001 phase I/II trial, the overall response rate to acalabrutinib, a highly selective BTK inhibitor, was 38% among DLBCL variant RS, the median PFS was 3 months and the median duration of response 5 months [62].

Constitutive AKT phosphorylation is significantly increased in high-risk CLL patients harboring *TP53* and *NOTCH1* mutations in comparison to wild-type patients. Furthermore, pAKT immunofluorescence showed increased expression and frequency in RS patients in comparison to both CLL and de novo DLBCL patients. Genetic over-activation of AKT in the murine Eμ-TCL1 CLL mouse model resulted in the transformation into high-grade lymphoma with phenotypic features of RS. Collectively, the data provide evidence that activation of AKT causes transformation of CLL into aggressive lymphoma [63••]. The PI3K inhibitor idelalisib showed some activity in patients with RS [64•]. These data prompt the investigation of PI3K inhibitors in this setting.

Since most of the DLBCL variant of RS show *TP53* disruption, novel drugs for this condition need to act independently of *TP53*. Venetoclax is a specific inhibitor of BCL2 that acts in a *TP53*-independent way and is effective in high-risk CLL [65]. Venetoclax is a specific inhibitor of BCL2 that acts with a TP53-independent mechanism and is effective in high-risk CLL. In the M12-175 (NCT01328626) phase I study, a limited number of DLBCL variant RS were treated with escalating doses of venetoclax, achieving a response rate of 43% (no CRs) [65]. The venetoclax-R-EPOCH combination was assessed in a phase 2 study in RS (NCT03054896) [66••]. Of the 21 evaluable patients who have started combination therapy, 16 responded (ORR 59%); 48% had CR, all of whom also showed undetectable bone marrow minimal residual disease (MRD) for the underlying CLL. With a median follow-up of 9 months, the reported median PFS and OS are both 16 months. Toxicities from intensive CIT and venetoclax were described including grade 3–4 neutropenia (58%), anemia (50%), thrombocytopenia (50%), and febrile neutropenia (38%). No tumor lysis syndrome (TLS) occurred with daily venetoclax ramp-up after 1 lead in cycle of R-EPOCH [66^••^].

The DLBCL variant of RS frequently occurs upon an exhausted immune system, due to immune checkpoint deregulation. This *scenario* includes expression of high levels of checkpoint inhibitory molecules (i.e., PD-1) on RS tumor cells. Pembrolizumab, an antibody that targets the PD-1 receptor, provided signals of activity in DLBCL variant RS (NCT02332980) [67]. Objective responses were observed in 44% (4/9) DLBCL variant RS patients. All responses were observed in patients with transformation after prior therapy with ibrutinib. The median OS of this cohort was 10.7 months but was not reached in DLBCL variant RS previously exposed to ibrutinib.

Synergistic antitumor effects between ibrutinib and inhibition of the PD-1 and PD-L1 pathway have been reported in preclinical studies [68]. The inhibition of interleukin 2-inducible T cell kinase, which plays a part in T cell proliferation and differentiation, might explain the role of ibrutinib in the modulation of the immune system. A phase 1/2a study was designed to assess the safety and efficacy of ibrutinib in combination with nivolumab in patients with relapsed or refractory hematological malignancies including high-risk CLL/SLL, follicular lymphoma, DLBCL, and RS [69••]. Overall response was seen in the Richter’s transformation cohort (13 [65%] of 20 patients), with two CRs and 11 PRs. The median duration of response was 6.9 months for the RS cohort.

Preliminary data on the administration of CAR-T cells in the setting of RS report discouraging responses (one disease progression, one evolution to PBL), but further studies are warranted [70, 71]. Whether the condition of the T cell pool can influence the proliferation of CAR-T cells has not been reported. Quantitative and qualitative impairment of immune system is observed in patients with CLL, including alterations of the innate immune system (i.e., defective function of neutrophils, natural killer (NK) cells, and decreased complement activity) and of the adaptive immune response (i.e., deficits in cell-mediated immunity with hypogammaglobulinemia, down-regulation of T cell function and defects in antibody dependent cellular cytotoxicity) [72]. In addition to impaired cytotoxicity and expansion, CAR-T cell exhaustion can lead to the failure of CAR-T cell therapy [73].

Results from a pilot study aiming at assessing the efficacy of concurrent ibrutinib through leukapheresis, lymphodepletion, and CD19 CAR-T cells infusion in heavily pretreated high-risk R/R CLL patients who had failed ibrutinib showed high response rates in all patients (4 patients with DLBCL variant CLL) with an ORR of 83%. Tolerability was acceptable, with most patients well tolerating the combination of ibrutinib and CD19 CAR-T cells, but caution is warranted in patients with CRS while receiving ibrutinib after CAR-T cell immunotherapy [74•].

## Conclusions and Future Perspectives

The recent genetic tools helped in understanding the molecular basis of RS and led to depict RS as a complex entity based on clonal and nonclonal evolutionary patterns, which impact on outcomes. Relapsed/refractory patients with CLL on novel agents are a new prognostic group with a potential adverse outcome when eventually experimenting transformation. Occurring mostly in elderly patients with different comorbidities, RS can have a limited treatment due to potential toxicities in this fragile population. Even if improved outcomes have been reported over the last 20 years (particularly after the introduction of rituximab), the outcome of RS patients is still poor.

The development of new preclinical models mimicking human RS may help in identifying new treatment targets and elaborating strategies for patients developing this aggressive disease. Early intervention policies for the high-risk CLL population might be explored. The trend of the increased use of novel agents versus standard CIT should likely prevent the selection of high-risk chemoresistant clones and the accumulation of genomic instability due to treatment toxicity.

An international and common effort in developing preclinical models, prognosticators, biobanks and databases should be pursued to improve outcomes in patients with RS.
